# Genome Sequence of “*Candidatus* Arthromitus” sp. Strain SFB-Mouse-NL, a Commensal Bacterium with a Key Role in Postnatal Maturation of Gut Immune Functions

**DOI:** 10.1128/genomeA.00705-14

**Published:** 2014-07-17

**Authors:** Alexander Bolotin, Tomas de Wouters, Pamela Schnupf, Christiane Bouchier, Valentin Loux, Moez Rhimi, Alexandre Jamet, Rozenn Dervyn, Samira Boudebbouze, Hervé M. Blottière, Alexei Sorokin, Johannes Snel, Nadine Cerf-Bensussan, Valérie Gaboriau-Routhiau, Maarten van de Guchte, Emmanuelle Maguin

**Affiliations:** a INRA, UMR1319 Micalis, Jouy en Josas, France; b AgroParisTech, UMR Micalis, Jouy en Josas, France; c INSERM UMR1163, Laboratory of Intestinal Immunity, Paris, France; d Université Paris Descartes-Sorbonne Paris Cité and Institut Imagine, Paris, France; e Institut Pasteur, Plate-forme Génomique, Paris, France; f INRA, UR1077 Mathématique Informatique et Génome, Jouy en Josas, France; g NIZO Food Research, Ede, The Netherlands

## Abstract

“*Candidatus* Arthromitus” sp. strain SFB-mouse-NL (SFB, segmented filamentous bacteria) is a commensal bacterium necessary for inducing the postnatal maturation of homeostatic innate and adaptive immune responses in the mouse gut. Here, we report the genome sequence of this bacterium, which sets it apart from earlier sequenced mouse SFB isolates.

## GENOME ANNOUNCEMENT

“*Candidatus* Arthromitus” spp., also known as segmented filamentous bacteria (SFB), are noncultivable, spore-forming, *Clostridia*-related commensal bacteria that colonize the digestive tracts of many animal species (1). SFB typically form filaments that are solidly anchored in gut epithelial cells (2), notably in the ileum. In mice, SFB play a key role in the postnatal maturation of gut innate and adaptive immune functions, and notably, they can induce a strong IgA response (3, 4) and the recruitment and activation of intraepithelial CD8^+^ T lymphocytes (5) and lamina propria CD4^+^ T cells (6, 7; reviewed in reference 8).

Here, we report the complete genome sequence of the mouse-specific isolate “*Candidatus* Arthromitus” sp. SFB-mouse-NL. While an earlier sequenced mouse SFB isolate from Japan was shown to elicit a T helper (Th) 17 immune response in the intestinal lamina propria of C57BL/6 mice (7), the present strain, an independent isolate from The Netherlands (9), was reported to not only induce a strong Th17 response but also to foster Th1, Th2, and regulatory T-cell responses in C3H/HeN mice (6). To define whether the observed differences may be related to genetic variations between the two SFB isolates, we determined the genome sequence of strain SFB-mouse-NL.

DNA was isolated from fecal material from SFB-monocolonized mice using a modified version of the protocol described in Morita et al. (10). The DNA was sequenced using Illumina paired-end sequencing technology. The sequence reads were filtered using CLC and BOWTIE (11) to eliminate mouse genome sequences, and they were assembled using CLC, ABySS (12), and SOAP*denovo* (13), yielding 40 scaffolds >1,000 bp with high BLASTn scoring to existing SFB genome sequences. Finishing was performed using GAP4 of the Staden package (14) through iterative selection of read pair mapping to scaffold extremities and independent *de novo* assembly of these reads by SOAP. The scaffolds were also ordered using Mauve aligner (15), with earlier published SFB genome sequences as the reference, and some sequence gaps bridged by PCR performed on the basis of this alignment and sequencing of the PCR products. Genome annotation was performed using RAST (16).

The genome of “*Candidatus* Arthromitus” sp. SFB-mouse-NL consists of one circular chromosome (1,654,902 bp) with 1,598 predicted coding sequences (CDS). The genome contains 4 (partial) prophages. A comparative analysis of the “*Candidatus* Arthromitus” sp. SFB-mouse-NL genome and two complete and 7 incomplete other SFB genomes retrieved from NCBI (http://www.ncbi.nlm.nih.gov/genomes/) using MUMmer (17) and Mauve (18) revealed that the SFB-mouse-NL genome is distinct (0.4% of overall nucleotide divergence) from the other genomes (which show <0.2% of overall nucleotide divergence among them).

### Nucleotide sequence accession number.

The genome sequence of “*Candidatus* Arthromitus” sp. SFB-mouse-NL has been deposited in GenBank under the accession no. CP008713.
